# Supplementary material for: Effects of Exercise-Based Rehabilitation on Lumbar Degenerative Disc Disease: A Systematic Review
Source: Healthcare (Basel). 2025 Aug 7;13(15):1938. doi: 10.3390/healthcare13151938 (PMC12346320; doi:10.3390/healthcare13151938)
Supplement: Supplementary file 1 [file healthcare-13-01938-s001.zip › healthcare-3782483-supplementary.pdf]

PRISMA 2020 for Abstracts Checklist

Section and Topic	Item #	Checklist item	Reported (Yes/No)
**TITLE**			
Title	1	Identify the report as a systematic review.	Yes
**BACKGROUND**			
Objectives	2	Provide an explicit statement of the main objective(s) or question(s) the review addresses.	Yes
**METHODS**			
Eligibility criteria	3	Specify the inclusion and exclusion criteria for the review.	Yes
Information sources	4	Specify the information sources (e.g. databases, registers) used to identify studies and the date when each was last searched.	Yes
Risk of bias	5	Specify the methods used to assess risk of bias in the included studies.	Yes
Synthesis of results	6	Specify the methods used to present and synthesise results.	Yes
**RESULTS**			
Included studies	7	Give the total number of included studies and participants and summarise relevant characteristics of studies.	Yes
Synthesis of results	8	Present results for main outcomes, preferably indicating the number of included studies and participants for each. If meta-analysis was done, report the summary estimate and confidence/credible interval. If comparing groups, indicate the direction of the effect (i.e. which group is favoured).	Yes
**DISCUSSION**			
Limitations of evidence	9	Provide a brief summary of the limitations of the evidence included in the review (e.g. study risk of bias, inconsistency and imprecision).	Yes
Interpretation	10	Provide a general interpretation of the results and important implications.	Yes
**OTHER**			
Funding	11	Specify the primary source of funding for the review.	No
Registration	12	Provide the register name and registration number.	Yes

From: Page MJ, McKenzie JE, Bossuyt PM, Boutron I, Hoffmann TC, Mulrow CD, et al. The PRISMA 2020 statement: an updated guideline for reporting systematic reviews. BMJ 2021;372:n71. doi: 10.1136/bmj.n71. This work is licensed under CC BY 4.0. To view a copy of this license, visit https://creativecommons.org/licenses/by/4.0/

PRISMA 2020 Checklist

Section and Topic	Item #	Checklist item	Location where item is reported
**TITLE**			
Title	1	Identify the report as a systematic review.	Page 1, Line 1-3
**ABSTRACT**			
Abstract	2	See the PRISMA 2020 for Abstracts checklist.	The PRISMA 2020 for Abstracts checklist file has been filled out separately and is available as a supplementary file.
**INTRODUCTION**			
Rationale	3	Describe the rationale for the review in the context of existing knowledge.	Page 3, Lines 92-109
Objectives	4	Provide an explicit statement of the objective(s) or question(s) the review addresses.	Page 3, Lines 109-114
**METHODS**			
Eligibility criteria	5	Specify the inclusion and exclusion criteria for the review and how studies were grouped for the syntheses.	Page 4, Lines 150-170
Information sources	6	Specify all databases, registers, websites, organisations, reference lists and other sources searched or consulted to identify studies. Specify the date when each source was last searched or consulted.	Page 3, Lines 128-133
Search strategy	7	Present the full search strategies for all databases, registers and websites, including any filters and limits used.	Page 3, Lines 134-142, Page 4, Lines 143-148
Selection process	8	Specify the methods used to decide whether a study met the inclusion criteria of the review, including how many reviewers screened each record and each report retrieved, whether they worked independently, and if applicable, details of automation tools used in the process.	Page 4, Lines 158-162, and Line 172-181
Data collection process	9	Specify the methods used to collect data from reports, including how many reviewers collected data from each report, whether they worked independently, any processes for obtaining or confirming data from study investigators, and if applicable, details of automation tools used in the process.	Page 4, Line 171-181
Data items	10a	List and define all outcomes for which data were sought. Specify whether all results that were compatible with each outcome domain in each study were sought (e.g. for all measures, time points, analyses), and if not, the methods used to decide which results to collect.	Page 4, Lines 162-170 (General description); Pages 12-14, Table 3(B) (Specific outcomes listed)
	10b	List and define all other variables for which data were sought (e.g. participant and intervention characteristics, funding sources). Describe any assumptions made about any missing or unclear information.	Page 4, Lines 171-181 (General description); Pages 8-12, Table 3(A) (Specific variables listed)
Study risk of bias assessment	11	Specify the methods used to assess risk of bias in the included studies, including details of the tool(s) used, how many reviewers assessed each study and whether they worked independently, and if applicable, details of automation tools used in the process.	Page 4, Lines 182-200

PRISMA 2020 Checklist

Section and Topic	Item #	Checklist item	Location where item is reported
Effect measures	12	Specify for each outcome the effect measure(s) (e.g. risk ratio, mean difference) used in the synthesis or presentation of results.	Not Reported
Synthesis methods	13a	Describe the processes used to decide which studies were eligible for each synthesis (e.g. tabulating the study intervention characteristics and comparing against the planned groups for each synthesis (item #5)).	Page 4, Lines 158-162 (Based on the overall eligibility criteria)
	13b	Describe any methods required to prepare the data for presentation or synthesis, such as handling of missing summary statistics, or data conversions.	Page 4, Lines 161-170
	13c	Describe any methods used to tabulate or visually display results of individual studies and syntheses.	Page 7, Line 271 to Page 14, Line 279. (Reference to tables); Pages 7-14, Tables 2 & 3.
	13d	Describe any methods used to synthesize results and provide a rationale for the choice(s). If meta-analysis was performed, describe the model(s), method(s) to identify the presence and extent of statistical heterogeneity, and software package(s) used.	Page 5, Lines 201-212
	13e	Describe any methods used to explore possible causes of heterogeneity among study results (e.g. subgroup analysis, meta-regression).	Page 5, Lines 201-212 (Heterogeneity was cited as a reason not to perform a meta-analysis, but methods to explore it were not described).
	13f	Describe any sensitivity analyses conducted to assess robustness of the synthesized results.	Not Reported
Reporting bias assessment	14	Describe any methods used to assess risk of bias due to missing results in a synthesis (arising from reporting biases).	Not Reported
Certainty assessment	15	Describe any methods used to assess certainty (or confidence) in the body of evidence for an outcome.	Not Reported
**RESULTS**			
Study selection	16a	Describe the results of the search and selection process, from the number of records identified in the search to the number of studies included in the review, ideally using a flow diagram.	Page 6, Lines 220-226 (Text) and Page 5, Figure 1 (Flow diagram).
	16b	Cite studies that might appear to meet the inclusion criteria, but which were excluded, and explain why they were excluded.	Page 3, Lines 161-162 (Reasons for exclusion are given, but specific studies are not cited).
Study characteristics	17	Cite each included study and present its characteristics.	Pages 7-14, Tables 3(A) & 3(B).
Risk of bias in studies	18	Present assessments of risk of bias for each included study.	Pages 7-8, Table 2.
Results of	19	For all outcomes, present, for each study: (a) summary statistics for each group (where appropriate) and (b) an effect estimate and its	Not Reported

PRISMA 2020 Checklist

Section and Topic	Item #	Checklist item	Location where item is reported
individual studies		precision (e.g. confidence/credible interval), ideally using structured tables or plots.	
Results of syntheses	20a	For each synthesis, briefly summarise the characteristics and risk of bias among contributing studies.	Page 4, Lines 183-200, Page 6, Line 232-236
	20b	Present results of all statistical syntheses conducted. If meta-analysis was done, present for each the summary estimate and its precision (e.g. confidence/credible interval) and measures of statistical heterogeneity. If comparing groups, describe the direction of the effect.	Page 5, Lines 201-212 (Presents the narrative synthesis, as no statistical synthesis was done).
	20c	Present results of all investigations of possible causes of heterogeneity among study results.	Not Reported
	20d	Present results of all sensitivity analyses conducted to assess the robustness of the synthesized results.	Not Reported
Reporting biases	21	Present assessments of risk of bias due to missing results (arising from reporting biases) for each synthesis assessed.	Not Reported
Certainty of evidence	22	Present assessments of certainty (or confidence) in the body of evidence for each outcome assessed.	Not Reported
**DISCUSSION**			
Discussion	23a	Provide a general interpretation of the results in the context of other evidence.	Page 14-16, Lines 279-376
	23b	Discuss any limitations of the evidence included in the review.	Page 16, Lines 345-358.
	23c	Discuss any limitations of the review processes used.	Page 14, Lines 377-403.
	23d	Discuss implications of the results for practice, policy, and future research.	Page 14-15, Lines 404-414.
**OTHER INFORMATION**			
Registration and protocol	24a	Provide registration information for the review, including register name and registration number, or state that the review was not registered.	Page 1, Lines 25-26 and Page 3, Lines 125-127.
	24b	Indicate where the review protocol can be accessed, or state that a protocol was not prepared.	The article protocol was registered in PROSPERO under registration number [CRD420251088811].
	24c	Describe and explain any amendments to information provided at registration or in the protocol.	Amendments to information provided at registration be accessible in PROSPERO under registration number CRD420251088811.
Support	25	Describe sources of financial or non-financial support for the review, and the role of the funders or sponsors in the review.	Page 17, Line 422.

PRISMA 2020 Checklist

Section and Topic	Item #	Checklist item	Location where item is reported
Competing interests	26	Declare any competing interests of review authors.	Page 17, Line 425.
Availability of data, code and other materials	27	Report which of the following are publicly available and where they can be found: template data collection forms; data extracted from included studies; data used for all analyses; analytic code; any other materials used in the review.	Not Reported

From: Page MJ, McKenzie JE, Bossuyt PM, Boutron I, Hoffmann TC, Mulrow CD, et al. The PRISMA 2020 statement: an updated guideline for reporting systematic reviews. BMJ 2021;372:n71. doi: 10.1136/bmj.n71.
This work is licensed under CC BY 4.0. To view a copy of this license, visit https://creativecommons.org/licenses/by/4.0/

Systematic Search Strategy for PubMed, Scopus, Web of Science and Google Scholar

Database	Search Date	Keywords/Search Syntax	Filters	Results
**PubMed**	January 25, 2025	("Intervertebral disc disease"[Supplementary Concept] OR "lumbar discopathy"[All Fields] OR "discogenic low back pain"[All Fields] OR "lumbar degenerative disc disease"[Title/Abstract] OR "lumbar discopathy"[Title/Abstract]) AND ("Exercise Movement Techniques"[MeSH Terms] OR "Aquatic Therapy"[MeSH Terms] OR "motion therapy, continuous passive"[MeSH Terms] OR "Massage"[MeSH Terms] OR "exercise therapy"[MeSH Terms] OR "Hydrotherapy"[MeSH Terms] OR "rehabilitation"[MeSH Terms] OR "Pilates"[Title/Abstract] OR "Yoga"[Title/Abstract] OR "Suspension training"[Title/Abstract] OR "Kinesiotaping"[Title/Abstract] OR "Motor Control Exercise"[Title/Abstract] OR "McKenzie"[Title/Abstract] OR "williams method"[Title/Abstract] OR ("physical therapy modalities"[MeSH Terms] OR ("physical"[All Fields] AND "therapy"[All Fields] AND "modalities"[All Fields]) OR "physical therapy modalities"[MeSH Terms])) AND (("english"[Language] OR "persian"[Language]) AND 2010/01/01:2025/01/01[Date - Publication])	Language: English, Persian<br><br>Publication Years: 2010–2025	64
**Scopus**	January 25, 2025	(TITLE-ABS-KEY ("Intervertebral disc disease" OR "lumbar discopathy" OR "discogenic low back pain" OR "lumbar degenerative disc disease" OR "DDD")) AND (TITLE-ABS-KEY ("Exercise" OR "Training" OR "Exercise Movement Techniques" OR "Aquatic Therapy" OR "motion therapy, continuous passive" OR "Massage" OR "exercise therapy" OR "Hydrotherapy" OR "rehabilitation" OR "Pilates" OR "Yoga" OR "Suspension training" OR "Kinesiotaping" OR "Motor Control Exercise" OR "McKenzie" OR "Williams")) AND (PUBYEAR > 2009 AND PUBYEAR < 2025) AND (LIMIT-TO (LANGUAGE , "English") OR LIMIT-TO (LANGUAGE , "Persian"))	Language: English/Persian, Publication Years: 2010–2025	304
**Web of Science**	January 25, 2025	(TS=("Intervertebral disc disease" OR "lumbar discopathy" OR "discogenic low back pain" OR "lumbar degenerative disc disease" OR "DDD")) AND (TS=("Exercise*" OR "Training*" OR "Exercise Movement Techniques" OR "Aquatic Therapy" OR "motion therapy, continuous passive" OR "Massage*" OR "exercise therapy" OR "Hydrotherapy*" OR "Rehabilitation*" OR "Pilates*" OR "Yoga*" OR "Suspension training" OR	Language: English, Publication Year: 2010–2025	227

		"Kinesiotaping" OR "Kinesiotape*"OR "Motor Control" OR "McKenzie" OR "Williams" OR "physical therapy")) AND (PY=(2010-2025)) AND (LA=(English OR Persian))		
**Google Scholar**	January 25, 2025	allintitle:("low back pain" OR "LBP" OR "discopathy" OR "disc herniation" OR "lumbar disc" OR "intervertebral disc disease" OR "DDD") AND ("Exercise" OR "Training" OR "Exercise Movement Techniques" OR "Aquatic Therapy" OR "motion therapy, continuous passive" OR "Massage" OR "exercise therapy" OR "Hydrotherapy" OR "rehabilitation" OR "Pilates" OR "Yoga" OR "Suspension training" OR "Kinesiotaping" OR "Motor Control Exercise" OR "McKenzie" OR "Williams"))	Manual filters: 2010–2025	~1900 (pre-screening)

Total: 2495
